# Clinical characteristics and risk factors for severe scrub typhus in pediatric and elderly patients

**DOI:** 10.1371/journal.pntd.0010357

**Published:** 2022-04-29

**Authors:** Xiu-Gang Guan, Yue-Hong Wei, Bao-Gui Jiang, Shi-Xia Zhou, An-Ran Zhang, Qing-Bin Lu, Zi-Wei Zhou, Jin-Jin Chen, Hai-Yang Zhang, Yang Ji, Yang Yang, Li-Qun Fang, Hao Li, Zhi-Cong Yang, Wei Liu

**Affiliations:** 1 State Key Laboratory of Pathogen and Biosecurity, Beijing Institute of Microbiology and Epidemiology, Beijing, China; 2 Guangzhou Center for Disease Control and Prevention, Guangzhou, China; 3 School of Public Health, Anhui Medical University, Hefei, China; 4 School of Public Health, Cheeloo College of Medicine, Shandong University, Jinan, China; 5 Department of Laboratorial Science and Technology, School of Public Health, Peking University, Beijing, China; 6 Department of Biostatistics, College of Public Health and Health Professions, and Emerging Pathogens Institute, University of Florida, Gainesville, Florida, United States of America; Seoul National University College of Medicine, REPUBLIC OF KOREA

## Abstract

**Background:**

Scrub typhus (ST) is a life-threatening infectious disease if appropriate treatment is unavailable. Large discrepancy of clinical severity of ST patients was reported among age groups, and the underlying risk factors for severe disease are unclear.

**Methods:**

Clinical and epidemiological data of ST patients were collected in 55 surveillance hospitals located in Guangzhou City, China, from 2012 to 2018. Severe prognosis and related factors were determined and compared between pediatric and elderly patients.

**Results:**

A total of 2,074 ST patients including 209 pediatric patients and 1,865 elderly patients were included, with a comparable disease severity rate of 11.0% (95% CI 7.1%–16.1%) and 10.3% (95% CI 9.0%–11.8%). Different frequencies of clinical characteristics including lymphadenopathy, skin rash, enlarged tonsils, etc. were observed between pediatric and elderly patients. Presence of peripheral edema and decreased hemoglobin were the most important predictors of severe illness in pediatric patients with adjusted ORs by 38.99 (9.96–152.67, p<0.001) and 13.22 (1.54–113.50, p = 0.019), respectively, while presence of dyspnea and increased total bilirubin were the potential determinants of severe disease in elderly patients with adjusted ORs by 11.69 (7.33–18.64, p<0.001) and 3.17 (1.97–5.11, p<0.001), respectively. Compared with pediatric patients, elderly patients were more likely to receive doxycycline (64.8% v.s 9.9%, p<0.001), while less likely to receive azithromycin therapy (5.0% v.s 41.1%, p<0.001).

**Conclusion:**

The disease severity rate is comparable between pediatric and elderly ST patients, while different clinical features and laboratory indicators were associated with development of severe complications for pediatric and elderly patients, which is helpful for diagnosis and progress assessment of disease for ST patients.

## Author summary

The study investigated different clinical features and risk factors for severe disease between pediatric and elderly patients with scrub typhus. We found that significantly higher frequencies of lymphadenopathy, skin rash, enlarged tonsils, etc. were observed in pediatric patients than elderly patients. Other non-specific signs, subjective complaints, and chest radiographic abnormality were overpresented in elderly patients. Risk factors for severe disease between pediatric and elderly patients with scrub typhus were different. Presence of peripheral edema and decreased hemoglobin were the most important factors for pediatric patients, while presence of dyspnea and increased total bilirubin for elderly patients. These findings reminded medical workers to acknowledge this important difference and to adopt an age specific method in the differential diagnosis and risk assessment for scrub typhus.

## Introduction

Scrub typhus (ST) caused by *Orientia tsutsugamushi* (*O*. *tsutsugamushi*), a systemic, life-threatening disease with an enormous incidence in Asia and the islands of the Pacific and Indian Oceans, remains largely neglected [1]. In recent years, the geographic distribution of ST has shown an expanding trend, with cases reported in Bhutan and Nepal in Asia, and areas outside the “tsutsugamushi triangle” such as the United Arab Emirates in the Middle East, Kenya in East Africa, and Peru and Chile in South America [2–8]. This has caused enhanced health concerns, especially under the situation of no vaccines available for prevention [9].

Generally, ST is associated with a broad range of symptoms from mild disease such as non-specific symptoms (i.e., fever, headache, myalgia, and cough), skin damage (i.e., eschar, ulcer, and skin rash), and gastrointestinal symptoms (i.e., nausea, vomiting, and abdominal pain), to severe complications including pneumonia, acute respiratory distress syndrome, acute kidney injury, myocarditis, pericarditis, meningitis, meningoencephalitis, and shock, etc [10–13]. If not appropriately treated, ST can result in severe multiple organ failure with a median series case fatality rate (CFR) up to 1.8% for children under 10 years of age and 29.4% for the elderly [14]. In a consistent manner, there had been increased susceptibility of pediatric and elderly patients to severe disease, evidenced by increased disease severity rate (DSR) than their counterparts, according to the most recent clinical studies [15–17]. Understanding the clinical aspects of ST disease among pediatric and elderly patients is critical for the sake of determining the high priority of fatal prevention, and aggressive therapeutic strategies, especially when considering the growing number of infants and the growing proportion elderly population in the worldwide range [18]. It might be unreasonable to use the same criteria to evaluate disease progression and prognosis in pediatric and elderly patients with ST. Although each group had been separately evaluated [15,17,19], no studies have ever assessed the difference between pediatric and elderly patients for their clinical manifestations and risk factors of severe disease, especially for those resided in the same endemic region and received appropriate treatment. This study aims to explore the similarities and differences of the clinical features and the underlying risk factors of severe disease between pediatric and elderly patients with ST by using a comprehensive and systematic review of medical records.

## Methods

### Ethics statement

The research protocol was approved by the Human Ethics Committee of Guangzhou Center for Disease Control and Prevention (GZCDC-ECHR-2020p0012).

### Study design and participants

This retrospective observational study was conducted from January 1, 2012, to December 31, 2018, in Guangzhou City, where the first human case with ST and the highest case number in China had been reported [20]. A total of 55 hospitals of all sizes located through all 11 districts, from large tertiary hospitals to small community hospitals in both rural and urban areas, that have ever reported ST cases, were included in the study. All clinically diagnosed or laboratory-confirmed ST patients that had been diagnosed and treated according to the guideline of the *National Scrub Typhus Control and Prevention Guideline (2009)* issued by the Chinese Center for Disease Control and Prevention were included in this study (S1 Table). Briefly, clinically diagnosed ST was defined as patients who had a history of field activities in endemic regions within the past three weeks and presented with fever and eschar/ulcer, or those who reported no clear exposure history but had the clinical presence of fever, eschar/ulcer, lymphadenopathy, and skin rash, during the epidemic season [21]. Laboratory-confirmed ST was defined as clinically diagnosed patients with one of the positive laboratory tests from the clinical samples: Weil-Felix test, indirect immune fluorescence antibody assay (IFA), polymerase chain reaction (PCR), or isolation of *O*. *tsutsugamushi* [21,22]. All ST patients have been excluded for other infectious diseases with similar clinical features that were likewise endemic in Guangzhou City, such as dengue fever, hemorrhagic fever with renal syndrome, and leptospirosis (S1 Fig).

### Data source

Data for this study were derived from the de-identified ST related database that had been established based on data from 55 hospitals in Guangzhou City. The data regarding patients’ demographic information, medical history, and exposure history were obtained by interviewing patients or their guardians. Clinical data during their whole hospitalization which comprised of the date of disease onset (when clinical signs or symptoms were noticed), clinical signs and symptoms, laboratory measurements, imaging findings, and treatment regimens were extracted from the medical records. All these data had been retrieved and proofread by a trained group of medical staff as previously described [23].

### Definition of patients and disease severity

According to the *National Bureau of Statistics*, pediatric patients were defined as age ≤14 years and elderly patients were defined as age ≥60 years. Severe ST patients were defined as those presenting with multiple organ dysfunction syndrome (MODS) or shock, or requiring intensive care unit (ICU) admission during their hospitalization for ST therapy, and mild ST patients were defined as those presenting none of these severe complications.

### Statistical analysis

Continuous variables were expressed as median (IQR, interquartile range). Categorical variables were summarized as frequencies or proportions. Chi-square test, Fisher’s exact test, or Mann-Whitney U test, were used as appropriate, to determine the inter-group difference. The risk factor analysis of severe disease was performed for pediatric and elderly patients separately. The clinical manifestation (reported before or on hospital admission) related variables with case numbers <5 and laboratory indicator (tested within 24 hours of admission) related variables with >50% missing value were excluded for the first step (S2 Fig). Thereafter, the Least Absolute Shrinkage and Selection Operator (LASSO) regression was performed with disease severity used as a binary outcome, the clinical manifestations and laboratory indicators used as independent variables separately, together with 5 demographic characteristics (S2 Fig). Finally, the remaining variables with model coefficients not zero selected by LASSO regression analysis were entered into a multivariate logistic regression analysis to assess their association with severe disease (S2 Fig). The R library “glmnet” was used for the LASSO regression. The odds ratio (OR) and the 95% confidence interval (95% CI) were estimated using maximum likelihood methods.

For the laboratory indicators that were significant in each multivariate logistic regression analysis, we delineated their dynamic pattern during the entire hospitalization for severe and mild patients separately by applying generalized estimating equation in the R library “geepack”.

A 2-sided p value of <0.05 is considered statistically significant. All statistical analyses were performed using R software (version 3.6.2, R Foundation).

## Results

### Baseline characteristics and clinical severity of patients with scrub typhus

Records of totally 5,274 hospitalized patients with ST in the dataset were screened, among whom 209 pediatric patients with median age of 5 years old (IQR, 2–8) and 1,865 elderly patients with median age of 66 years old (IQR, 62–72) were included for analysis (Table 1 and S1 Fig). A total of 263 patients were laboratory-confirmed by Weil-Felix test (157 cases), IFA (66 cases), PCR (35 cases), and both Weil-Felix test and IFA or PCR (5 cases) (S2 Table). Most variables were comparable between clinical diagnostic cases and laboratory-confirmed cases for pediatric and elderly patients (S3 Table). Significantly more males were observed in the pediatric patients than elderly patients (57.9% v.s 36.7%, p<0.001) and more pediatric patients were recorded from urban areas than elderly patients (45.5% v.s 25.7%, p<0.001). The median (IQR) time from symptom onset to hospital admission in pediatric patients was 8 (6–10) days, significantly longer than that in elderly patients (7, 4–9 days, p<0.001) (Table 1). Severe illness was determined from 11.0% (95% CI 7.1%–16.1%) of pediatric patients and 10.3% (95% CI 9.0%–11.8%) of elderly patients, yielding comparable disease severity rate (DSR) between two groups (Table 1). Moreover, within the elderly group, we observed a significantly increased DSR as age increased, with the highest DSR observed for patients ≥80 years (17.4%) (S4 Table). No age-specific trend of DSR was observed among the pediatric group (S4 Table). In both groups, MODS was the predominant complication (10.0% for pediatric patients and 7.1% for elderly patients respectively), followed by ICU admission and shock (Table 1).

**Table 1:** Demographic and clinical characteristics compared between pediatric and elderly patients with scrub typhus.

	All patients (n = 2,074)	Pediatric patients (n = 209)	Elderly patients (n = 1,865)	p value*
Age, years, median (IQR)	65 (61–71)	5 (2–8)	66 (62–72)	NA
Sex				<0.001
Female	1,269 (61.2)	88 (42.1)	1,181 (63.3)	
Male	805 (38.8)	121 (57.9)	684 (36.7)	
Residence				<0.001
Urban	574 (27.7)	95 (45.5)	479 (25.7)	
Rural	1,500 (72.3)	114 (54.5)	1,386 (74.3)	
Time from symptom onset to hospital admission, days, median (IQR)				
	7 (4–10)	8 (6–10)	7 (4–9)	<0.001^‡^
Length of hospital stay, days, median (IQR)				
	8 (6–11)	8 (7–10)	8 (6–11)	0.112^‡^
Severe cases				
	216 (10.4)	23 (11.0)	193 (10.3)	0.861
Severe complications				
MODS	153 (7.4)	21 (10.0)	132 (7.1)	0.156
ICU admission	114 (5.5)	14 (6.7)	100 (5.4)	0.520
Shock	86 (4.1)	11 (5.3)	75 (4.0)	0.502
Outcome				0.251
Death	35 (1.7)	1 (0.5)	34 (1.8)	
Survived	2,039 (98.3)	208 (99.5)	1,831 (98.2)	

Data are n (%) until otherwise indicated. NA, not applicable.

Pediatric patients, age 0–14 years; elderly patients, age ≥60 years.

*p value calculated by use of χ^2^ test or Fisher’s exact test between pediatric patients and elderly patients.

^‡^p value calculated by Mann-Whitney U test between pediatric patients and elderly patients.

IQR, interquartile range; MODS, multiple organ dysfunction syndrome; ICU, intensive care unit.

### Clinical manifestations and the association with severe disease

**Fig 1:**
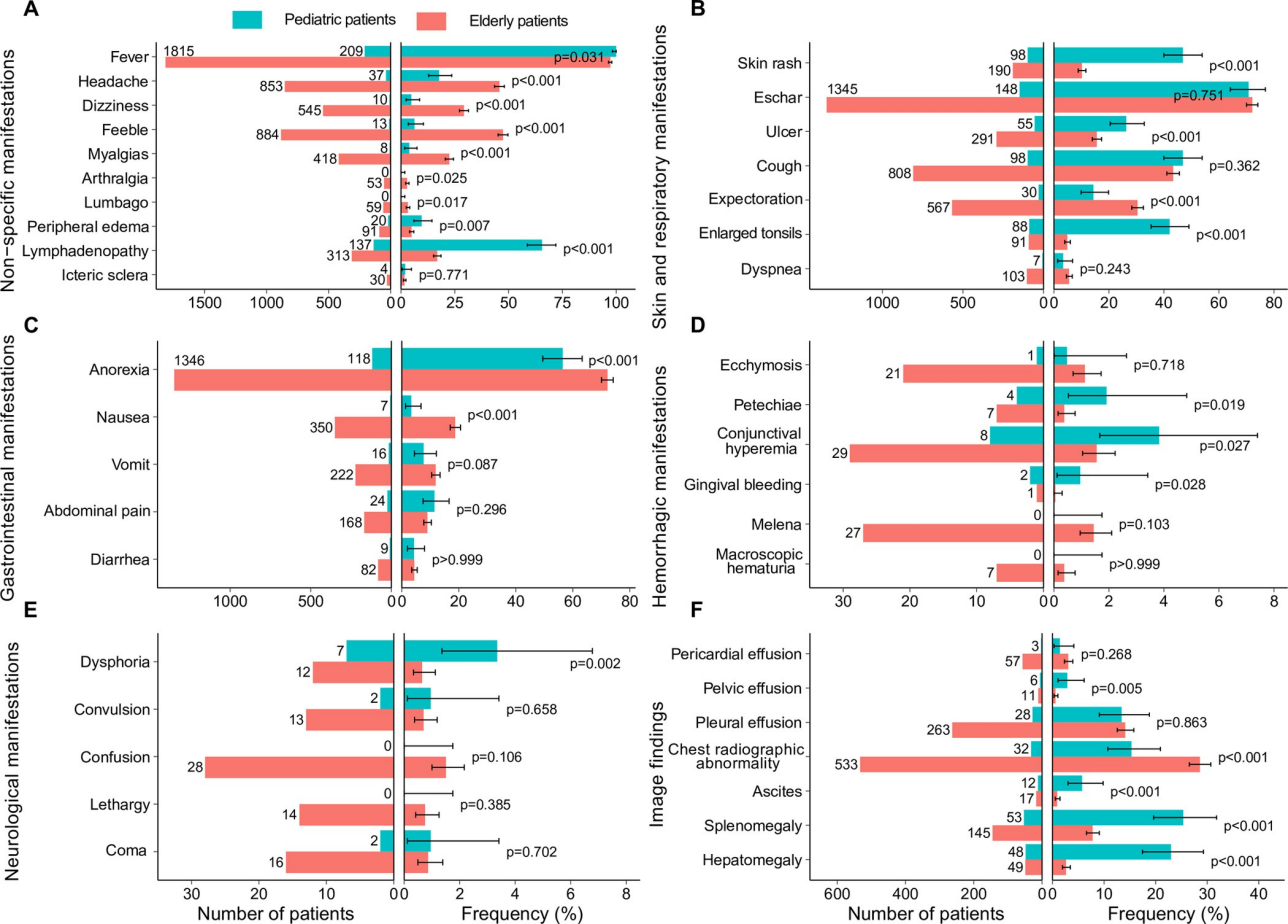
The clinical characteristics compared between pediatric and elderly patients with scrub typhus disease in Guangzhou, China. The case number and frequency of patients who reported each of the pre-designed clinical manifestations were presented for pediatric group (0–14 years) and elderly group (≥60 years) patients separately. The clinical manifestations were grouped into non–specific manifestations (A), skin and respiratory manifestations (B), gastrointestinal manifestations (C), hemorrhagic manifestations (D), neurological manifestations (E), and image findings (F). p value indicated comparison of frequencies between pediatric and elderly patients for each pre-designed clinical manifestation by use of χ^2^ test or Fisher’s exact test.

The frequencies of clinical symptoms differed between two age groups. Compared with elderly patients, pediatric patients were featured by significantly higher frequencies of lymphadenopathy (65.6% v.s 16.8%), skin rash (46.9% v.s 10.2%), enlarged tonsils (42.1% v.s 4.9%), ulcer (26.3% v.s 15.6%) and peripheral edema (9.6% v.s 4.9%) which were identified through physical examination, together with significantly higher frequencies of splenomegaly (25.4% v.s 7.8%), hepatomegaly (23.0% v.s 2.6%), ascites (5.7% v.s 0.9%) and pelvic effusion (2.9% v.s 0.6%) which were identified through imaging examination (Fig 1 and S5 Table). In contrast, chest radiographic abnormality was more frequently recorded among elderly patients than pediatric patients (28.6% v.s 15.3%), other non-specific signs and subjective complaints that were overpresented in elderly patients included anorexia (72.2% v.s 56.5%), headache (45.7% v.s 17.7%), feeble (47.4% v.s 6.2%), dizziness (29.2% v.s 4.8%), myalgias (22.4% v.s 3.8%), and nausea (18.8% v.s 3.3%) (Fig 1 and S5 Table).

**Fig 2:**
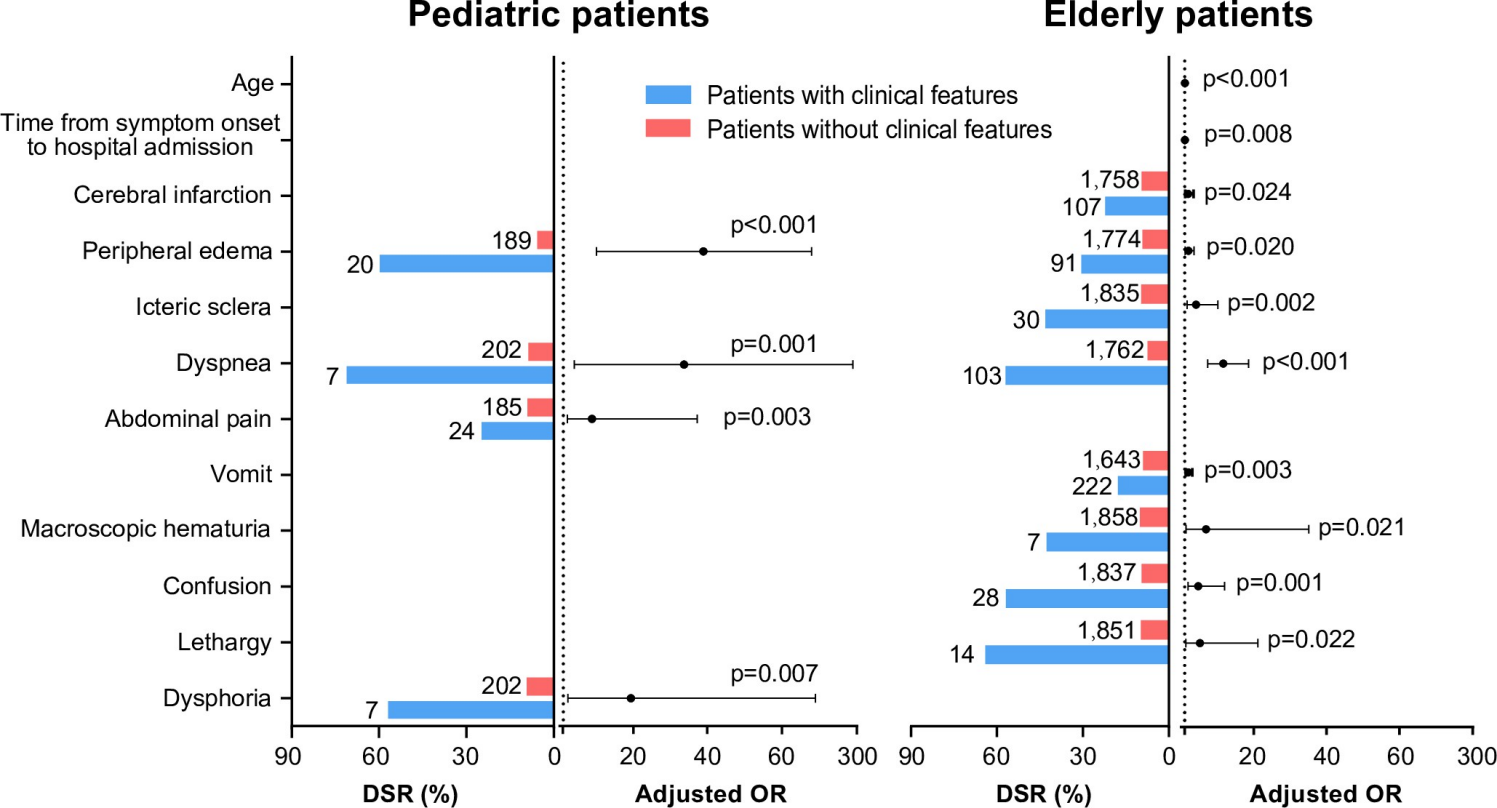
The major clinical symptoms and signs in association with disease severity rates in patients with scrub typhus. The number of patients with or without each of clinical symptoms/signs is shown at the left of the columns, based on which the disease severity rate was calculated and marked by the length of the column. The black points indicate the adjusted ORs for disease severity by using multivariate logistic regression model; the black error bars indicate the 95% confidence intervals. The dotted line indicates an adjusted OR of 1. DSR, disease severity rate; OR, odds ratio. Pediatric patients, age 0–14 years; elderly patients, age ≥60 years.

Multivariate logistic regression analysis disclosed differential clinical manifestations that were significantly related to severe ST in two age groups. Peripheral edema (adjusted OR 38.99, 95% CI 9.96–152.67), dyspnea (adjusted OR 33.71, 95% CI 3.95–287.53), dysphoria (adjusted OR 19.30, 95% CI 2.26–165.07), and abdominal pain (adjusted OR 8.80, 95% CI 2.08–37.27) were significantly related to severe diseases for pediatric patients (Fig 2 and S6 Table). While dyspnea (adjusted OR 11.69, 95% CI 7.33–18.64), macroscopic hematuria (adjusted OR 6.89, 95% CI 1.34–35.32), lethargy (5.19, 95% CI 1.27–21.27), confusion (adjusted OR 4.75, 95% CI 1.87–12.04), icteric sclera (adjusted OR 4.13, 95% CI 1.67–10.19), peripheral edema (adjusted OR 2.00, 95% CI 1.11–3.60), vomit (adjusted OR 1.98, 95% CI 1.27–3.11), preexisting cerebral infarction (adjusted OR 1.94, 95% CI 1.09–3.45), time from symptom onset to hospital admission (adjusted OR 1.06, 95% CI 1.02–1.11) and age (adjusted OR 1.05, 95% CI 1.03–1.07) were significant for elderly patients. Only peripheral edema and dyspnea were significant among both groups (Fig 2 and S6 Table).

### Laboratory indicators and the association with severe disease

A total of 15 laboratory indicators (8 hematological and 7 biochemical) were tested on hospital admission, from which 5 were abnormal among over 50% of pediatric patients, and 6 were abnormal among over 50% of elderly patients (S7 Table). The top five indicators with abnormal measurement in pediatric patients included elevated level of C reactive protein (CRP) (145/182, 79.7%), alanine aminotransferase (ALT) (102/186, 54.8%), decreased albumin (ALB) (152/171, 88.9%), mean corpuscular volume (MCV) (144/193, 74.6%), and platelet (PLT) count (108/194, 55.7%). This differed from elderly patients for whom the top five abnormalities were elevated CRP (1,123/1,277, 87.9%), ALT (1,246/1,717, 72.6%), neutrophil (NEU) percent (1,071/1,694, 63.2%), decreased hematocrit (HCT) (1,279/1,718, 74.4%), and ALB (1,236/1,708, 72.4%) (S7 Table).

Multivariate logistic regression analysis disclosed different laboratory abnormalities that were significantly related to severe ST between the two groups. The significant factors included decreased hemoglobin (HGB) (adjusted OR 13.22, 95% CI 1.54–113.5), increased ALT (adjusted OR 10.53, 95% CI 1.14–97.48), decreased PLT count (adjusted OR 6.03, 95% CI 1.01–36.03), and increased total bilirubin (TBIL) (adjusted OR 6.40, 95% CI 1.17–34.99) for pediatric patients (S9 Table). More significant factors were demonstrated in elderly patients yet with lower effects than the pediatric patients, including increased TBIL (adjusted OR 3.17, 95% CI 1.97–5.11), decreased PLT count (adjusted OR 3.15, 95% CI 1.92–5.16), decreased HGB (adjusted OR 2.84, 95% CI 1.76–4.60), and increased creatinine (CREA) (adjusted OR 2.44, 95% CI 1.50–3.95) were disclosed (S9 Table). It’s notable that only decreased PLT count, HGB, and increased TBIL were significant among both groups (S9 Table).

**Fig 3:**
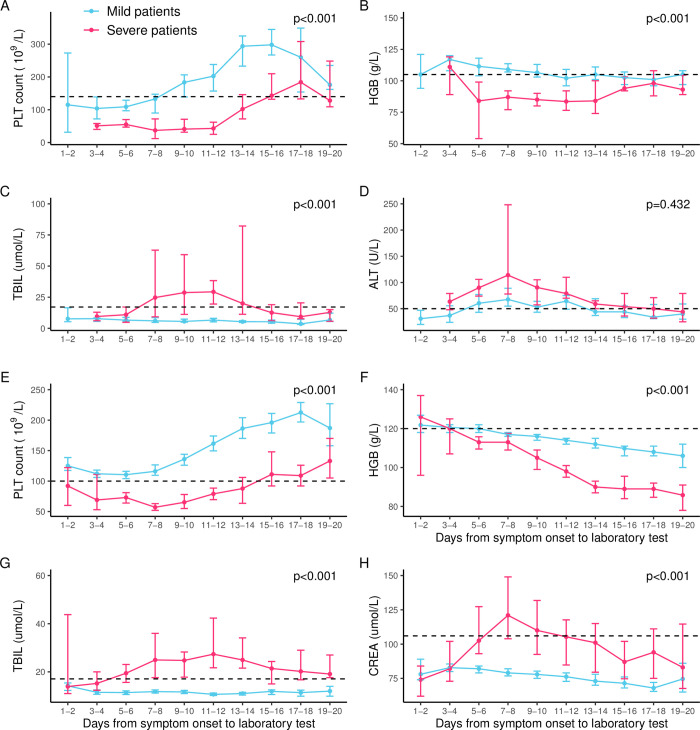
The dynamic pattern of major laboratory indicators associated with severe disease was delineated along the days from symptom onset by applying GEE for pediatric patients (A–D) and elderly patients (E–H). Four significant laboratory indicators in pediatric patients (age 0–14 years) were delineated: PLT count (A), HGB (B), TBIL(C), and ALT (D). Four significant laboratory indicators in elderly patients (age ≥60 years) were delineated: PLT count (E), HGB (F), TBIL(G), and CREA (H). The median value was shown by point, and 95% confidence interval was shown in the error bars. Normal ranges for each variable are 140–440 ×10^9^/L for PLT count, 105–145 g/L for HGB, 5.1–17.1 umol/L for TBIL, and 0–50 U/L for ALT in pediatric patients, and 100–300 ×10^9^/L for PLT, 120–165 g/L for HGB, 5.1–17.1 umol/L for TBIL, and 53–106 umol/L for CREA in elderly patients. GEE, generalized estimating equation; PLT, platelet; HGB, hemoglobin; TBIL, total bilirubin; ALT, alanine aminotransferase; CREA, creatinine.

The dynamic pattern of the laboratory indicators with significant effect in each multivariate logistic regression model was delineated for severe and mild patients. Among pediatric patients, the PLT count at early disease was below the normal in both groups and with even lower level and delayed recovery to normal in the severe group compared with the mild group (at 15–16 and 9–10 days after disease onset respectively) (Fig 3A). HGB reached its nadir at 5–6 days and lasted for about 8 days before it starts to rise, but did not reach the normal level till the last observation point in severe patients (Fig 3B). TBIL remained at plateau level for the mild patients under the normal range, which however peaked at 9–10 days post disease onset and return to normal level at two weeks for the severe patients (Fig 3C). For elderly patients, all the evaluations of PLT, TBIL, and CREA were maintained within the normal range in the mild group, while in the severe group, their abnormalities reached the greatest extent at 7–8, 11–12, and 7–8 days respectively (Fig 3E, 3G, and 3H). Noticeably, HGB decreased in a consistent manner without recovery till the last observation in both groups, and shown with a significantly lower level in severe patients than in mild patients (Fig 3F). With exception of ALT in pediatric patients, all the major laboratory indicators were significantly different between severe and mild patients during the entire hospitalization by using generalized estimating equation (S10 Table).

### Treatments and clinical outcomes

Drug use data were obtained from 202 (96.7%) of pediatric patients, among them, 95.5% of patients received antibiotics treatment, including azithromycin in 83 (41.1%), chloramphenicol in 40 (19.8%), doxycycline in 20 (9.9%), and other antibiotics (erythrocin and roxithromycin) in 17 (8.4%), and combined administration of over 2 antibiotics in 33 (16.3%) of the patients (S11 Table). Drug use data were obtained from 1,735 (93.0%) of elderly patients and 98.2% received antibiotics treatment, revealing that elderly patients were more likely to receive doxycycline therapy (64.8% v.s 9.9%, p<0.001) as compared with pediatric patients, while less likely to receive azithromycin therapy than the pediatric patients (5.0% v.s 41.1%, p<0.001) (S11 Table). The same regimens of combined antibiotics therapy were administered with comparable frequencies between pediatric patients and elderly patients (16.3% v.s 13.1%, p = 0.250) (S11 Table). The median length of hospital stay was 8 days (IQR, 7–10) for pediatric patients, which did not differ from elderly patients (median, 8 days; IQR, 6–11 days; p = 0.112) (Table 1). Totally thirty-five of the severe cases died, with the CFR in pediatric patients lower than that of the elderly, yet with no statistical significance attained (0.5%, 95% CI 0.0%–2.6% v.s 1.8%, 95% CI 1.3%–2.5%; p = 0.251) (Table 1).

## Discussion

This retrospective study, to our knowledge, is the first study to present age differences in clinical characteristics among severe ST patients, and the risk factors for the development of severe ST as well. Our multivariate analysis revealed that pediatric and elderly patients presented differential clinical manifestations that were related to severe ST, which provide important reference for clinical diagnosis and prognosis assessment of ST.

Despite of an exhaustive collection of all complications, including MODS, shock, and ICU admission, as the principal causes of death, we found the currently determined severe complications in pediatric and elderly patients were less frequent than those of previous studies [15–17], most likely due to the antibiotic treatment that had been widely applied for the current patients. Discrepancies regarding the clinical phenotype and risk factors related to the adverse outcomes were also obvious between pediatric and elderly ST patients. Specifically, all of the abnormalities except for eschar that were determined from physical examination were more commonly seen among pediatric than among elderly patients, by contrast, the non-specific clinical complaints were less frequently reported from pediatric patients, which situation was due to their limited language skills. Higher frequencies of lymphadenopathy (65.6% v.s 23.0%; p<0.001) and neurological symptoms (4.8% v.s 1.7%; p = 0.020) were observed in children in the current study compared with previously reported data [24,25]. Chest radiographic abnormality, which used to be considered as the common condition of severe ST, was more likely to be recorded from elderly patients than general population (28.6% v.s 23.6%, p<0.001) [26], reflecting the high susceptibility to pneumonia in this group due to physiological dysfunctions and increased susceptibility to infections in old subjects [27].

Common laboratory abnormalities of ST included elevated liver transaminases, thrombocytopenia, and hypoalbuminemia. Thrombocytopenia and decreased MCV could serve as more specific indicators for diagnostic purposes in pediatric patients, in contrast, the elevated liver transaminases were more specific in elderly patients. These findings, taken together indicated the necessity of adopting age-stratified analysis when the clinical diagnosis was made. For example, to make diagnosis among pediatric patients, indicators such as lymphadenopathy, splenomegaly, hepatomegaly, and skin rash, should be used with higher priority than eschar. Still, all these signs are non-specific and resemble other tropical infections like malaria, typhoid, dengue, or leptospirosis [28].

Another noteworthy finding was that peripheral edema and dyspnea were commonly related to the severe disease for both pediatric and elderly patients, with the more robust effect seen from pediatric patients, highlighting their critical role in the disease outcome. As a pathophysiological hallmark of ST [29], disseminated vasculitis can occur with subsequent vascular injury involving skin, liver, brain, kidney, and lung, etc. Vascular endothelial permeability may increase systemically and lead to pathologic capillary leak syndrome resulting in tissue peripheral edema and intravascular hypovolemia [30]. This can explain the prominent role of peripheral edema, as well as the decreased albumin indicative of liver injury. Decreased levels of PLT count, surrogates of capillary permeability, were highly associated with severe disease in both pediatric and elderly patients. This was consistent with the clinical data showing decreased PLT count and HGB are more common in pediatric patients than in elderly patients. The dynamic pattern of laboratory indicators reveals that HGB has been decreasing and not returning to normal levels in patients with severe and mild diseases. This is related to the pre-existing condition of unnoticed anemia which was related to high susceptibility to the severe form of the disease and aggravated the clinical condition further. There were previous studies that reported anemia in association with increased severity of ST [31,32] and this association was reinforceed in an independent group of patients in our study. In previous studies, hypoalbuminemia was a risk fator of disease severity [17,33]. However, hypoalbuminemia was commonly observed in pediatric (88.9%) and elderly patients (72.4%) with ST in the present study, suggesting this indicator might be less specific in associated with severe illness. In addition, neutrophilia that was recognized as a disease severity factor [34], was not identified in our data. Abdominal pain is the main gastrointestinal manifestation in ST patients, and in a study of upper gastrointestinal endoscopy in ST patients, was present in 9.8% (25/256) of patients [35]. As a risk factor for severe ST in pediatric patients, which is associated with a sensitive and underdeveloped gastrointestinal system, which has a greater chance of leading to small vascular lesions in the gastrointestinal mucosa and consequently to severe illness.

As is known to all, *O*. *tsutsugamushi* has been widely reported to invade the central nervous system and cause encephalitis [13,36,37]. In our study, the comorbidity of cerebral infarction was a risk factor for severe disease, and cerebral infarction was a serious cerebrovascular disease with focal ischemic brain tissue necrosis. Patients with underlying cerebral infarction further aggravate their neurological damage and thus are more likely to develop severe disease or death post infection of *O*. *tsutsugamushi*.

This study is subject to several limitations. There are uncontrollable confounding factors for a large-scale study with long time span and multi-hospitals. Only hospitalized patients were included for analysis, hence outpatients who were not admitted to the hospital warrant further investigation. Only a part of analysed ST patients were confirmed by laboratory diagnosis, and those who only meet clinically diagnosis criteria might deviate from the laboratory-confirmed ones. Other limitations include missing or incomplete data on subjective complaints among children under 5 years such as sore throat, arthralgia, myalgia, or headache, which were not applied in the logistic regression model.

In summary, our study illustrated different clinical features and laboratory indicators that were associated with development of severe complications for pediatric and elderly patients. Within endemic regions, it is essential for medical workers to acknowledge this important difference and to adopt an age specific method in the differential diagnosis and risk assessment for ST, especially under circumstances of persistent challenges in the diagnosis of ST in resource-limited settings.

## Supporting information

S1 Table Diagnosis criteria and classification for scrub typhus according to the *National Scrub Typhus Control and Prevention Guideline (2009)*. (DOCX)

S2 Table Number of patients with positive test in different diagnostic methods. IFA: indirect immunofluorescence antibody assay; PCR, polymerase chain reaction.
(DOCX)

S3 Table The differences between clinical diagnostic cases and laboratory-confirmed cases in pediatric and elderly patients with scrub typhus. Data are n (%) until otherwise indicated. NA, not applicable. Pediatric patients, age 0–14 years; elderly patients, age ≥60 years. p value* calculated by use of χ^2^ test or Fisher’s exact test between clinical diagnostic cases and laboratory-confirmed cases. p value^‡^ calculated by Mann-Whitney U test between clinical diagnostic cases and laboratory-confirmed cases. IQR, interquartile range; COPD, chronic obstructive pulmonary disease. WBC, white blood cells; PLT, platelet; HGB, hemoglobin; LYM, lymphocyte; NEU, neutrophil; MON, monocyte; HCT, hematocrit; MCV, mean corpuscular volume; TBIL, total bilirubin; ALT, alanine aminotransferase; ALB, albumin; GLB, globulin; CREA, creatinine; BUN, blood urea nitrogen; CRP, C reactive protein.
(DOCX)

S4 Table The disease severity rate of scrub typhus patients among age groups of pediatric and elderly patients. DSR, disease severity rate. Age groups of pediatric patients, years^§^: infants (age 0–3 years), preschool children (age 4–6 years), and school-age children (age 7–14 years).
(DOCX)

S5 Table Different clinical characteristics between pediatric and elderly patients with scrub typhus. Data are n (%). NA, not applicable. Pediatric patients, age 0–14 years; elderly patients, age ≥60 years. All clinical symptoms (non-specific, skin, respiratory, gastrointestinal, hemorrhagic, and neurological) were reported before or on hospital admission, all abnormal image findings were reported after hospital admission. p value calculated by use of χ^2^ test or Fisher’s exact test between pediatric and elderly patients. COPD, chronic obstructive pulmonary disease. Hemorrhagic manifestations*, patients with one or more hemorrhagic symptoms. Neurological manifestations*, patients with one or more neurological symptoms.
(DOCX)

S6 Table Association between clinical characteristics and severe scrub typhus by multivariate logistic regression analysis for pediatric and elderly patients. NA, not applicable. The non-significant association was left blank. OR, odds ratio. CI, confidence interval. Pediatric patients, age 0–14 years; elderly patients, age ≥60 years.
(DOCX)

S7 Table The laboratory abnormalities on hospital admission compared between pediatric and elderly patients with scrub typhus. Data was no./No. (%), no. is the number of patients with presence of laboratory abnormality. No. is the total number of patients with available data. Ranks of laboratory abnormalities according to their frequencies were listed for two age subgroups separately. Pediatric patients, age 0–14 years; elderly patients, age ≥60 years. p value calculated for the difference between pediatric patients and elderly patients by use of χ^2^ test or Fisher’s exact test. The normal range of laboratory indicators was expressed as that of the normal adult male. Normal ranges in different sex and age were shown in S8 Table. WBC, white blood cells; PLT, platelet; HGB, hemoglobin; LYM, lymphocyte; NEU, neutrophil; MON, monocyte; HCT, hematocrit; MCV, mean corpuscular volume; TBIL, total bilirubin; ALT, alanine aminotransferase; ALB, albumin; GLB, globulin; CREA, creatinine; BUN, blood urea nitrogen; CRP, C reactive protein.
(DOCX)

S8 Table Normal ranges of laboratory indicators by sex and age. WBC, white blood cells; PLT, platelet; HGB, hemoglobin; LYM, lymphocyte; NEU, neutrophil; MON, monocyte; HCT, hematocrit; MCV, mean corpuscular volume; TBIL, total bilirubin; ALT, alanine aminotransferase; ALB, albumin; GLB, globulin; CREA, creatinine; BUN, blood urea nitrogen; CRP, C reactive protein.
(DOCX)

S9 Table Association between laboratory measurements and severe scrub typhus by multivariate logistic regression analysis for pediatric and elderly patients. The non-significant association was left blank. OR, odds ratio; PLT, platelet; HGB, hemoglobin; TBIL, total bilirubin; ALT, alanine aminotransferase; CREA, creatinine; CI, confidence interval. Pediatric patients, age 0–14 years; elderly patients, age ≥60 years. The normal range of laboratory indicators was expressed as that of the normal adult male. Different normal ranges in different sex and age were shown in S8 Table.
(DOCX)

S10 Table Estimated coefficients and statistical significance of major laboratory indicators associated with severe disease for pediatric patients and elderly patients during the days from symptom onset to laboratory test by using generalized estimating equation. NA, not applicable. Coefficients were estimated that how many units of increase or decrease for major laboratory indicators in severe patients from symptom onset to laboratory test with mild patients group used as reference. PLT, platelet; HGB, hemoglobin; TBIL, total bilirubin; ALT, alanine aminotransferase; CREA, creatinine; CI, confidence interval. Pediatric patients, age 0–14 years; elderly patients, age ≥60 years.
(DOCX)

S11 Table Therapy types for pediatric and elderly patients. Data are n (%). Other antibiotics included minocycline, levofloxacin, ciprofloxacin, erythrocin and roxithromycin, etc.
(DOCX)

S1 Fig The flowchart of recruiting and grouping of patients with scrub typhus. ST, scrub typhus. HFRS, hemorrhagic fever with renal syndrome. Pediatric patients, age 0–14 years; elderly patients, age ≥60 years.
(TIF)

S2 Fig Variable selection and model construction of the logistic regression for pediatric and elderly patients. Demographic characteristics (age, sex, time from symptom onset to hospital admission, and residence). Clinical manifestations (hypertension, diabetes, coronary heart disease, cerebral infarction, viral hepatitis, COPD, smoking, drinking, fever, headache, dizziness, feeble, myalgias, arthralgia, lumbago, peripheral edema, lymphadenopathy, icteric sclera, skin rash, eschar, ulcer, cough, expectoration, enlarged tonsils, dyspnea, anorexia, nausea, vomit, abdominal pain, diarrhea, ecchymosis, petechiae, conjunctival hyperemia, gingival bleeding, melena, macroscopic hematuria, dysphoria, convulsion, confusion, lethargy, and coma). Laboratory parameters (WBC count, PLT count, HGB, LYM percent, NEU percent, MON percent, HCT, MCV, TBIL, ALT, ALB, GLB, CREA, BUN, and CRP). Variables were excluded if patients with clinical manifestations <5 cases: 21 clinical manifestations were excluded for pediatric patients (hypertension, diabetes, coronary heart disease, cerebral infarction, viral hepatitis, COPD, smoking, drinking, arthralgia, lumbago, icteric sclera, ecchymosis, petechiae, gingival bleeding, melena, macroscopic hematuria, convulsion, confusion, lethargy, coma, and fever was also excluded as present in all pediatric patients); gingival bleeding was excluded for elderly patients. BUN was excluded if missing values >50% in pediatric and elderly patients. COPD, chronic obstructive pulmonary disease. residence, area type of patients’ residence (rural or urban). Pediatric patients, age 0–14 years; elderly patients, age ≥60 years. WBC, white blood cells; PLT, platelet; HGB, hemoglobin; LYM, lymphocyte; NEU, neutrophil; MON, monocyte; HCT, hematocrit; MCV, mean corpuscular volume; TBIL, total bilirubin; ALT, alanine aminotransferase; ALB, albumin; GLB, globulin; CREA, creatinine; BUN, blood urea nitrogen; CRP, C reactive protein.
(TIF)

S1 Data The numerical data used in Figs 2 and 3. (XLSX)
