# Supplementary material for: Clinical characteristics and risk factors for severe scrub typhus in pediatric and elderly patients
Source: PLoS Negl Trop Dis. 2022 Apr 29;16(4):e0010357. doi: 10.1371/journal.pntd.0010357 (PMC9053809; doi:10.1371/journal.pntd.0010357)
Supplement: S1 Table — (DOCX) [file pntd.0010357.s001.docx]

**S1** **Table:** **Diagnosis criteria and classification for scrub typhus according to the *National Scrub Typhus Control and Prevention Guideline (2009)*.**

|  | **National Scrub Typhus Control and Prevention Guideline. 2009** |
| --- | --- |
| **Epidemiology linkage** | 1.1 An individual who experienced possible outdoor exposure to mite bites three weeks before the onset of illness, i.e., farming, fishing, camping, and straw collection, during the epidemic season of the disease |
| **Clinical description** | 2.1 Fever  2.2 Lymphadenopathy 2.3 Skin rash  2.4 Specific eschars/ulcers |
| **Laboratory tests** | 3.1 An agglutination titer ≥1:160 in the Weil-Felix test using the OXK strain of *Proteus mirabilis*.  3.2 Seroconversion or a four-fold or greater rise in serum IgG antibody titers between acute and convalescent sera detected by using mixed Gilliam, Karp, Kato, and Kawasaki strains of *O. tsutsugamushi* as diagnostic antigen in indirect immunofluorescence antibody assay (IFA).  3.3 The detection of *O. tsutsugamushi* 56-kDa gene by polymerase chain reaction in clinical specimens.  3.4 The isolation of *O. tsutsugamushi* from clinical specimens. |
| **Diagnosis and classification** | Suspected case:  (1) a patient with item 1.1, 2.1, plus either 2.2 or 2.3, and was excluded from other diseases. Or   (2) a patient with item 2.1, 2.2 and 2.3 during the local epidemic season of scrub typhus (May–  November south of Yangtze River and October–November in north of Yangtze River).  Clinical diagnostic case:   (1) a suspected case with item 2.4. Or   (2) a patient with item of 1.1, 2.1, and 2.4.  Laboratory-confirmed case:   (1) a clinical diagnostic case with any one of the items 3.1–3.4. Or   (2) a suspected case with any one of the items 3.2–3.4 |
