# Supplementary material for: Clinical characteristics and risk factors for severe scrub typhus in pediatric and elderly patients
Source: PLoS Negl Trop Dis. 2022 Apr 29;16(4):e0010357. doi: 10.1371/journal.pntd.0010357 (PMC9053809; doi:10.1371/journal.pntd.0010357)
Supplement: S2 Table — IFA: indirect immunofluorescence antibody assay; PCR, polymerase chain reaction. (DOCX) [file pntd.0010357.s002.docx]

**S2** **Table: Number of patients with positive test in** **different diagnostic methods.**

| Diagnostic methods | Number of patients with positive test (n=263) |
| --- | --- |
| Weil-Felix test | 157 |
| IFA | 66 |
| PCR | 35 |
| Weil-Felix test & IFA | 3 |
| Weil-Felix test & PCR | 2 |
| Isolation of *O. tsutsugamushi* | 0 |

IFA: indirect immunofluorescence antibody assay; PCR, polymerase chain reaction.
