# Supplementary material for: Clinical characteristics and risk factors for severe scrub typhus in pediatric and elderly patients
Source: PLoS Negl Trop Dis. 2022 Apr 29;16(4):e0010357. doi: 10.1371/journal.pntd.0010357 (PMC9053809; doi:10.1371/journal.pntd.0010357)
Supplement: S3 Table — Data are n (%) until otherwise indicated. NA, not applicable. Pediatric patients, age 0–14 years; elderly patients, age ≥60 years. p value* calculated by use of χ2 test or Fisher’s exact test between clinical diagnostic cases and laboratory-confirmed cases. p value‡ calculated by Mann-Whitney U test between clinical diagnostic cases and laboratory-confirmed cases. IQR, interquartile range; COPD, chronic obstructive pulmonary disease. WBC, white blood cells; PLT, platelet; HGB, hemoglobin; LYM, lymphocyte; NEU, neutrophil; MON, monocyte; HCT, hematocrit; MCV, mean corpuscular volume; TBIL, total bilirubin; ALT, alanine aminotransferase; ALB, albumin; GLB, globulin; CREA, creatinine; BUN, blood urea nitrogen; CRP, C reactive protein. (DOCX) [file pntd.0010357.s003.docx]

**S3 Table: The differences** **between clinical diagnostic cases and laboratory-confirmed cases in pediatric and elderly patients with scrub typhus.**

| **Variables** | **Pediatric patients (N=209)** | | |  | **Elderly patients (n=1,865)** | | |
| --- | --- | --- | --- | --- | --- | --- | --- |
|  | **Laboratory-confirmed cases** | | **p value^*^** |  | **Laboratory-confirmed cases** | | **p value^*^** |
|  | **Yes (n=56)** | **No (n=153)** |  |  | **Yes (n=207)** | **No (n=1,658)** |  |
| Age, years, median (IQR) | 5 (2–8) | 4 (2–7) | 0.821^‡^ |  | 66 (63–72) | 66 (62–72) | 0.417^‡^ |
| Sex, male | 38 (67.9) | 83 (54.2) | 0.108 |  | 79 (38.2) | 605 (36.5) | 0.693 |
| Residence, rural | 23 (41.1) | 91 (59.5) | 0.027 |  | 129 (62.3) | 1257 (75.8) | <0.001 |
| Time from symptom onset to hospital admission, days, median (IQR) | | | |  |  |  |  |
|  | 8 (5–10) | 8 (6–10) | 0.178^‡^ |  | 7 (4–9) | 7 (4–10) | 0.098^‡^ |
| Length of hospital stay, days, median (IQR) | | | |  |  |  |  |
|  | 8 (6–10) | 9 (7–11) | 0.025^‡^ |  | 8 (6–11) | 8 (5.5–11) | 0.577^‡^ |
| Comorbidities |  |  |  |  |  |  |  |
| Hypertension | 0 (0) | 0 (0) | NA |  | 53 (25.6) | 464 (28.0) | 0.523 |
| Diabetes | 0 (0) | 0 (0) | NA |  | 29 (14.0) | 195 (11.8) | 0.409 |
| Coronary heart disease | 0 (0) | 0 (0) | NA |  | 11 (5.3) | 77 (4.6) | 0.799 |
| Cerebral infarction | 0 (0) | 0 (0) | NA |  | 18 (8.7) | 89 (5.4) | 0.075 |
| Viral hepatitis | 0 (0) | 0 (0) | NA |  | 4 (1.9) | 46 (2.8) | 0.632 |
| COPD | 0 (0) | 0 (0) | NA |  | 4 (1.9) | 38 (2.3) | >0.999 |
| Lifestyles |  |  |  |  |  |  |  |
| Smoking | NA | NA | NA |  | 34 (16.4) | 234 (14.1) | 0.430 |
| Drinking | NA | NA | NA |  | 13 (6.3) | 126 (7.6) | 0.588 |
| Non-specific manifestations | |  |  |  |  |  |  |
| Fever | 51 (100.0) | 158 (100.0) | NA |  | 202 (97.6) | 1613 (97.3) | 0.982 |
| Headache | 11 (19.6) | 26 (17.0) | 0.810 |  | 103 (49.8) | 750 (45.2) | 0.247 |
| Dizziness | 3 (5.4) | 7 (4.6) | 0.730 |  | 49 (23.7) | 496 (29.9) | 0.075 |
| Feeble | 3 (5.4) | 10 (6.5) | >0.999 |  | 87 (42.0) | 797 (48.1) | 0.117 |
| Myalgias | 3 (5.4) | 5 (3.3) | 0.444 |  | 61 (29.5) | 357 (21.5) | 0.013 |
| Arthralgia | 0 (0) | 0 (0) | NA |  | 7 (3.4) | 46 (2.8) | 0.784 |
| Lumbago | 0 (0) | 0 (0) | NA |  | 7 (3.4) | 52 (3.1) | >0.999 |
| Peripheral edema | 7 (12.5) | 13 (8.5) | 0.545 |  | 8 (3.9) | 83 (5.0) | 0.584 |
| Lymphadenopathy | 41 (73.2) | 96 (62.7) | 0.213 |  | 43 (20.8) | 270 (16.3) | 0.126 |
| Icteric sclera | 1 (1.8) | 3 (2.0) | >0.999 |  | 4 (1.9) | 26 (1.6) | 0.568 |
| Skin manifestations |  |  |  |  |  |  |  |
| Skin rash | 28 (50.0) | 70 (45.8) | 0.698 |  | 24 (11.6) | 166 (10.0) | 0.557 |
| Eschar/Ulcer | 49 (87.5) | 134 (87.6) | >0.999 |  | 175 (84.5) | 1381 (83.3) | 0.722 |
| Eschar | 39 (69.6) | 109 (71.2) | 0.957 |  | 152 (73.4) | 1193 (72.0) | 0.716 |
| Ulcer | 14 (25.0) | 41 (26.8) | 0.933 |  | 32 (15.5) | 259 (15.6) | >0.999 |
| Respiratory manifestations |  |  |  |  |  |  |  |
| Cough | 24 (42.9) | 74 (48.4) | 0.582 |  | 82 (39.6) | 726 (43.8) | 0.285 |
| Expectoration | 10 (17.9) | 20 (13.1) | 0.515 |  | 58 (28.0) | 509 (30.7) | 0.478 |
| Enlarged tonsils | 24 (42.9) | 64 (41.8) | >0.999 |  | 11 (5.3) | 80 (4.8) | 0.891 |
| Dyspnea | 2 (3.6) | 5 (3.3) | >0.999 |  | 10 (4.8) | 93 (5.6) | 0.764 |
| Gastrointestinal manifestations |  |  |  |  |  |  |  |
| Anorexia | 31 (55.4) | 87 (56.9) | 0.971 |  | 147 (71.0) | 1199 (72.3) | 0.755 |
| Nausea | 1 (1.8) | 6 (3.9) | 0.677 |  | 41 (19.8) | 309 (18.6) | 0.755 |
| Vomit | 4 (7.1) | 12 (7.8) | >0.999 |  | 22 (10.6) | 200 (12.1) | 0.626 |
| Abdominal pain | 7 (12.5) | 17 (11.1) | 0.973 |  | 23 (11.1) | 145 (8.7) | 0.321 |
| Diarrhea | 0 (0) | 9 (5.9) | 0.117 |  | 11 (5.3) | 71 (4.3) | 0.615 |
| Hemorrhagic manifestations^*^ | 5 (8.9) | 8 (5.2) | 0.340 |  | 13 (6.3) | 74 (4.5) | 0.320 |
| Ecchymosis | 0 (0) | 1 (0.6) | >0.999 |  | 4 (1.9) | 17 (1.0) | 0.281 |
| Petechiae | 3 (5.4) | 1 (0.7) | 0.060 |  | 1 (0.5) | 6 (0.4) | 0.562 |
| Conjunctival hyperemia | 1 (1.8) | 7 (4.6) | 0.685 |  | 5 (2.4) | 24 (1.4) | 0.363 |
| Gingival bleeding | 1 (1.8) | 1 (0.7) | 0.465 |  | 0 (0) | 1 (0.1) | >0.999 |
| Melena | 0 (0) | 0 (0) | NA |  | 4 (1.9) | 23 (1.4) | 0.532 |
| Macroscopic hematuria | 0 (0) | 0 (0) | NA |  | 0 (0) | 7 (0.4) | >0.999 |
| Neurological manifestations^*^ | 4 (7.1) | 6 (3.9) | 0.463 |  | 9 (4.3) | 54 (3.3) | 0.538 |
| Dysphoria | 2 (3.6) | 5 (3.3) | >0.999 |  | 2 (1.0) | 10 (0.6) | 0.634 |
| Convulsion | 1 (1.8) | 1 (0.7) | 0.465 |  | 1 (0.5) | 12 (0.7) | >0.999 |
| Confusion | 0 (0) | 0 (0) | NA |  | 2 (1.0) | 26 (1.6) | 0.762 |
| Lethargy | 0 (0) | 0 (0) | NA |  | 1 (0.5) | 13 (0.8) | >0.999 |
| Coma | 2 (3.6) | 0 (0) | 0.071 |  | 3 (1.4) | 13 (0.8) | 0.410 |
| Image findings |  |  |  |  |  |  |  |
| Pericardial effusion | 1 (1.8) | 2 (1.3) | >0.999 |  | 8 (3.9) | 49 (3.0) | 0.615 |
| Pelvic effusion | 1 (1.8) | 5 (3.3) | >0.999 |  | 0 (0) | 11 (0.7) | 0.623 |
| Pleural effusion | 9 (16.1) | 19 (12.4) | 0.647 |  | 44 (21.3) | 219 (13.2) | 0.002 |
| Chest radiographic abnormality | 11 (19.6) | 21 (13.7) | 0.404 |  | 60 (29.0) | 473 (28.5) | 0.956 |
| Ascites | 5 (8.9) | 7 (4.6) | 0.311 |  | 0 (0) | 17 (1.0) | 0.245 |
| Splenomegaly | 18 (32.1) | 35 (22.9) | 0.236 |  | 24 (11.6) | 121 (7.3) | 0.041 |
| Hepatomegaly | 14 (25.0) | 34 (22.2) | 0.813 |  | 9 (4.3) | 40 (2.4) | 0.158 |
| Hematological indicators, median (IQR) | |  |  |  |  |  |  |
| WBC count (×10^9^/L) | 7.9  (5.6–11.1) | 9.5  (5.9–12.5) | 0.225^‡^ |  | 7.1  (5.4–9.5) | 7.3  (5.1–9.4) | 0.520^‡^ |
| PLT count (×10^9^/L) | 141.1  (71.3–204.2) | 101.5  (62.2–179.8) | 0.083^‡^ |  | 115.0  (78.0–164.0) | 118.5  (79.2–166.8) | 0.927^‡^ |
| HGB (g/L) | 107.0  (93.5–119.0) | 104.5  (96.3–116.0) | 0.515^‡^ |  | 118.0  (108.0–128.0) | 118.5  (108.0–128.0) | 0.746^‡^ |
| LYM percent (%) | 37.9  (25.8–51.8) | 38.6  (28.0–50.7) | 0.613^‡^ |  | 17.5  (11.1–28.6) | 18.9  (11.6–30.6) | 0.322^‡^ |
| NEU percent (%) | 51.0  (35.4–64.0) | 52.5  (40.8–62.8) | 0.998^‡^ |  | 75.5  (61.8–82.9) | 74.0  (62.9–83.5) | 0.857^‡^ |
| MON percent (%) | 6.8  (4.9–9.0) | 6.0  (4.0–8.2) | 0.313^‡^ |  | 6.0  (4.1–8.3) | 6.0  (4.1–8.8) | 0.661^‡^ |
| HCT (%) | 31.8  (28.2–35.1) | 31.0  (27.9–34.2) | 0.211^‡^ |  | 35.0  (32.0–38.2) | 35.3  (32.4–38.3) | 0.471^‡^ |
| MCV (FL) | 79.0  (75.0–82.2) | 78.1  (75.9–80.9) | 0.314^‡^ |  | 87.0  (82.6–90.8) | 87.2  (82.0–90.7) | 0.965^‡^ |
| Biochemical indicators, median (IQR) | |  |  |  |  |  |  |
| TBIL (umol/L) | 6.7  (5.0–10.8) | 6.6  (4.8–8.8) | 0.371^‡^ |  | 12.0  (8.6–17.2) | 11.7  (8.7–16.6) | 0.416^‡^ |
| ALT (U/L) | 53.0  (34.0–90.0) | 65.0  (39.0–96.0) | 0.531^‡^ |  | 59.0  (38.3–91.0) | 57.0  (40.0–84.0) | 0.478^‡^ |
| ALB (g/L) | 34.8  (29.6–37.9) | 30.3  (26.9–35.3) | 0.004^‡^ |  | 31.7  (27.7–35.2) | 31.1  (27.5–36.1) | 0.835^‡^ |
| GLB (g/L) | 25.7  (22.7–30.4) | 26.9  (22.4–30.0) | 0.932^‡^ |  | 28.5  (25.4–32.0) | 28.9  (25.9–31.9) | 0.844^‡^ |
| CREA (umol/L) | 39.0  (30.3–52.3) | 38.0  (29.1–47.8) | 0.585^‡^ |  | 82.0  (67.0–103.8) | 80.0  (67.5–102.0) | 0.581^‡^ |
| BUN (mmol/L) | 3.2  (2.7–4.0) | 3.3  (2.5–4.2) | >0.999^‡^ |  | 5.1  (3.8–7.2) | 5.1  (3.9–6.6) | 0.809^‡^ |
| CRP (mg/L) | 30.8  (7.8–61.9) | 23.9  (7.5–58.1) | 0.800^‡^ |  | 51.9  (17.5–97.9) | 58.6  (23.1–98.6) | 0.721^‡^ |

Data are n (%) until otherwise indicated. NA, not applicable.

Pediatric patients, age 0–14 years; elderly patients, age ≥60 years.

p value^*^ calculated by use of χ^2^ test or Fisher's exact test between clinical diagnostic cases and laboratory-confirmed cases.

p value^‡^ calculated by Mann-Whitney U test between clinical diagnostic cases and laboratory-confirmed cases.

IQR, interquartile range; COPD, chronic obstructive pulmonary disease. WBC, white blood cells; PLT, platelet; HGB, hemoglobin; LYM, lymphocyte; NEU, neutrophil; MON, monocyte; HCT, hematocrit; MCV, mean corpuscular volume; TBIL, total bilirubin; ALT, alanine aminotransferase; ALB, albumin; GLB, globulin; CREA, creatinine; BUN, blood urea nitrogen; CRP, C reactive protein.

**Table S4: The disease severity rate of scrub typhus patients among age groups of pediatric and elderly patients.**

| **Age groups** | **Total cases** | **Severe cases** | **Mild cases** | **DSR (%)** | **p value** |
| --- | --- | --- | --- | --- | --- |
| Age groups of pediatric patients, years^§^, n (%) |  |  |  |  | 0.881 |
| 0–3 | 89 | 11 | 78 | 12.4 |  |
| 4–6 | 41 | 4 | 37 | 9.8 |  |
| 7–14 | 79 | 8 | 71 | 10.1 |  |
| Age groups of elderly patients, years, n (%) |  |  |  |  | 0.001 |
| 60–69 | 1,226 | 106 | 1,120 | 8.6 |  |
| 70–79 | 490 | 61 | 429 | 12.4 |  |
| ≥80 | 149 | 26 | 123 | 17.4 |  |

DSR, disease severity rate.

Age groups of pediatric patients, years^§^: infants (age 0–3 years), preschool children (age 4–6 years), and school-age children (age 7–14 years).

**Table S5: Different** **clinical characteristics between pediatric and elderly patients with scrub typhus.**

| **Characteristics** | **Pediatric patients**  **(n=209)** | **Elderly patients**  **(n=1,865)** | **p value** |
| --- | --- | --- | --- |
| Comorbidities |  |  |  |
| Hypertension | 0 (0) | 517 (27.7) | <0.001 |
| Diabetes | 0 (0) | 224 (12.0) | <0.001 |
| Coronary heart disease | 0 (0) | 88 (4.7) | 0.002 |
| Cerebral infarction | 0 (0) | 107 (5.7) | <0.001 |
| Viral hepatitis | 0 (0) | 50 (2.7) | 0.031 |
| COPD | 0 (0) | 42 (2.3) | 0.018 |
| Lifestyles |  |  |  |
| Smoking | NA | 268 (14.4) | NA |
| Drinking | NA | 139 (7.5) | NA |
| Non-specific manifestations | |  |  |
| Fever | 209 (100.0) | 1,815 (97.3) | 0.031 |
| Headache | 37 (17.7) | 853 (45.7) | <0.001 |
| Dizziness | 10 (4.8) | 545 (29.2) | <0.001 |
| Feeble | 13 (6.2) | 884 (47.4) | <0.001 |
| Myalgias | 8 (3.8) | 418 (22.4) | <0.001 |
| Arthralgia | 0 (0) | 53 (2.8) | 0.025 |
| Lumbago | 0 (0) | 59 (3.2) | 0.017 |
| Peripheral edema | 20 (9.6) | 91 (4.9) | 0.007 |
| Lymphadenopathy | 137 (65.6) | 313 (16.8) | <0.001 |
| Icteric sclera | 4 (1.9) | 30 (1.6) | 0.771 |
| Skin manifestations | |  |  |
| Skin rash | 98 (46.9) | 190 (10.2) | <0.001 |
| Eschar/Ulcer | 183 (87.6) | 1,556 (83.4) | 0.150 |
| Eschar | 148 (70.8) | 1,345 (72.1) | 0.751 |
| Ulcer | 55 (26.3) | 291 (15.6) | <0.001 |
| Respiratory manifestations | |  |  |
| Cough | 98 (46.9) | 808 (43.3) | 0.362 |
| Expectoration | 30 (14.4) | 567 (30.4) | <0.001 |
| Enlarged tonsils | 88 (42.1) | 91 (4.9) | <0.001 |
| Dyspnea | 7 (3.3) | 103 (5.5) | 0.243 |
| Gastrointestinal manifestations | |  |  |
| Anorexia | 118 (56.5) | 1,346 (72.2) | <0.001 |
| Nausea | 7 (3.3) | 350 (18.8) | <0.001 |
| Vomit | 16 (7.7) | 222 (11.9) | 0.087 |
| Abdominal pain | 24 (11.5) | 168 (9.0) | 0.296 |
| Diarrhea | 9 (4.3) | 82 (4.4) | >0.999 |
| Hemorrhagic manifestations^*^ | 13 (6.2) | 87 (4.7) | 0.409 |
| Ecchymosis | 1 (0.5) | 21 (1.1) | 0.718 |
| Petechiae | 4 (1.9) | 7 (0.4) | 0.019 |
| Conjunctival hyperemia | 8 (3.8) | 29 (1.6) | 0.027 |
| Gingival bleeding | 2 (1.0) | 1 (0.1) | 0.028 |
| Melena | 0 (0) | 27 (1.4) | 0.103 |
| Macroscopic hematuria | 0 (0) | 7 (0.4) | >0.999 |
| Neurological manifestations^*^ | 10 (4.8) | 63 (3.4) | 0.396 |
| Dysphoria | 7 (3.3) | 12 (0.6) | 0.002 |
| Convulsion | 2 (1.0) | 13 (0.7) | 0.658 |
| Confusion | 0 (0) | 28 (1.5) | 0.106 |
| Lethargy | 0 (0) | 14 (0.8) | 0.385 |
| Coma | 2 (1.0) | 16 (0.9) | 0.702 |
| Image findings | |  |  |
| Pericardial effusion | 3 (1.4) | 57 (3.1) | 0.268 |
| Pelvic effusion | 6 (2.9) | 11 (0.6) | 0.005 |
| Pleural effusion | 28 (13.4) | 263 (14.1) | 0.863 |
| Chest radiographic abnormality | 32 (15.3) | 533 (28.6) | <0.001 |
| Ascites | 12 (5.7) | 17 (0.9) | <0.001 |
| Splenomegaly | 53 (25.4) | 145 (7.8) | <0.001 |
| Hepatomegaly | 48 (23.0) | 49 (2.6) | <0.001 |

Data are n (%). NA, not applicable.

Pediatric patients, age 0–14 years; elderly patients, age ≥60 years.

All clinical symptoms (non-specific, skin, respiratory, gastrointestinal, hemorrhagic, and neurological) were reported before or on hospital admission, all abnormal image findings were reported after hospital admission.

p value calculated by use of χ^2^ test or Fisher's exact test between pediatric and elderly patients.

COPD, chronic obstructive pulmonary disease.

Hemorrhagic manifestations^*^, patients with one or more hemorrhagic symptoms.

Neurological manifestations^*^, patients with one or more neurological symptoms.
