# Supplementary material for: Clinical characteristics and risk factors for severe scrub typhus in pediatric and elderly patients
Source: PLoS Negl Trop Dis. 2022 Apr 29;16(4):e0010357. doi: 10.1371/journal.pntd.0010357 (PMC9053809; doi:10.1371/journal.pntd.0010357)
Supplement: S4 Table — DSR, disease severity rate. Age groups of pediatric patients, years§: infants (age 0–3 years), preschool children (age 4–6 years), and school-age children (age 7–14 years). (DOCX) [file pntd.0010357.s004.docx]

**S4 Table: The disease severity rate of scrub typhus patients among age groups of pediatric and elderly patients.**

| **Age groups** | **Total cases** | **Severe cases** | **Mild cases** | **DSR (%)** | **p value** |
| --- | --- | --- | --- | --- | --- |
| Age groups of pediatric patients, years^§^, n (%) |  |  |  |  | 0.881 |
| 0–3 | 89 | 11 | 78 | 12.4 |  |
| 4–6 | 41 | 4 | 37 | 9.8 |  |
| 7–14 | 79 | 8 | 71 | 10.1 |  |
| Age groups of elderly patients, years, n (%) |  |  |  |  | 0.001 |
| 60–69 | 1,226 | 106 | 1,120 | 8.6 |  |
| 70–79 | 490 | 61 | 429 | 12.4 |  |
| ≥80 | 149 | 26 | 123 | 17.4 |  |

DSR, disease severity rate.

Age groups of pediatric patients, years^§^: infants (age 0–3 years), preschool children (age 4–6 years), and school-age children (age 7–14 years).
