# Supplementary material for: Clinical characteristics and risk factors for severe scrub typhus in pediatric and elderly patients
Source: PLoS Negl Trop Dis. 2022 Apr 29;16(4):e0010357. doi: 10.1371/journal.pntd.0010357 (PMC9053809; doi:10.1371/journal.pntd.0010357)
Supplement: S5 Table — Data are n (%). NA, not applicable. Pediatric patients, age 0–14 years; elderly patients, age ≥60 years. All clinical symptoms (non-specific, skin, respiratory, gastrointestinal, hemorrhagic, and neurological) were reported before or on hospital admission, all abnormal image findings were reported after hospital admission. p value calculated by use of χ2 test or Fisher’s exact test between pediatric and elderly patients. COPD, chronic obstructive pulmonary disease. Hemorrhagic manifestations*, patients with one or more hemorrhagic symptoms. Neurological manifestations*, patients with one or more neurological symptoms. (DOCX) [file pntd.0010357.s005.docx]

**S5 Table: Different** **clinical characteristics between pediatric and elderly patients with scrub typhus.**

| **Characteristics** | **Pediatric patients**  **(n=209)** | **Elderly patients**  **(n=1,865)** | **p value** |
| --- | --- | --- | --- |
| Comorbidities |  |  |  |
| Hypertension | 0 (0) | 517 (27.7) | <0.001 |
| Diabetes | 0 (0) | 224 (12.0) | <0.001 |
| Coronary heart disease | 0 (0) | 88 (4.7) | 0.002 |
| Cerebral infarction | 0 (0) | 107 (5.7) | <0.001 |
| Viral hepatitis | 0 (0) | 50 (2.7) | 0.031 |
| COPD | 0 (0) | 42 (2.3) | 0.018 |
| Lifestyles |  |  |  |
| Smoking | NA | 268 (14.4) | NA |
| Drinking | NA | 139 (7.5) | NA |
| Non-specific manifestations | |  |  |
| Fever | 209 (100.0) | 1,815 (97.3) | 0.031 |
| Headache | 37 (17.7) | 853 (45.7) | <0.001 |
| Dizziness | 10 (4.8) | 545 (29.2) | <0.001 |
| Feeble | 13 (6.2) | 884 (47.4) | <0.001 |
| Myalgias | 8 (3.8) | 418 (22.4) | <0.001 |
| Arthralgia | 0 (0) | 53 (2.8) | 0.025 |
| Lumbago | 0 (0) | 59 (3.2) | 0.017 |
| Peripheral edema | 20 (9.6) | 91 (4.9) | 0.007 |
| Lymphadenopathy | 137 (65.6) | 313 (16.8) | <0.001 |
| Icteric sclera | 4 (1.9) | 30 (1.6) | 0.771 |
| Skin manifestations | |  |  |
| Skin rash | 98 (46.9) | 190 (10.2) | <0.001 |
| Eschar/Ulcer | 183 (87.6) | 1,556 (83.4) | 0.150 |
| Eschar | 148 (70.8) | 1,345 (72.1) | 0.751 |
| Ulcer | 55 (26.3) | 291 (15.6) | <0.001 |
| Respiratory manifestations | |  |  |
| Cough | 98 (46.9) | 808 (43.3) | 0.362 |
| Expectoration | 30 (14.4) | 567 (30.4) | <0.001 |
| Enlarged tonsils | 88 (42.1) | 91 (4.9) | <0.001 |
| Dyspnea | 7 (3.3) | 103 (5.5) | 0.243 |
| Gastrointestinal manifestations | |  |  |
| Anorexia | 118 (56.5) | 1,346 (72.2) | <0.001 |
| Nausea | 7 (3.3) | 350 (18.8) | <0.001 |
| Vomit | 16 (7.7) | 222 (11.9) | 0.087 |
| Abdominal pain | 24 (11.5) | 168 (9.0) | 0.296 |
| Diarrhea | 9 (4.3) | 82 (4.4) | >0.999 |
| Hemorrhagic manifestations^*^ | 13 (6.2) | 87 (4.7) | 0.409 |
| Ecchymosis | 1 (0.5) | 21 (1.1) | 0.718 |
| Petechiae | 4 (1.9) | 7 (0.4) | 0.019 |
| Conjunctival hyperemia | 8 (3.8) | 29 (1.6) | 0.027 |
| Gingival bleeding | 2 (1.0) | 1 (0.1) | 0.028 |
| Melena | 0 (0) | 27 (1.4) | 0.103 |
| Macroscopic hematuria | 0 (0) | 7 (0.4) | >0.999 |
| Neurological manifestations^*^ | 10 (4.8) | 63 (3.4) | 0.396 |
| Dysphoria | 7 (3.3) | 12 (0.6) | 0.002 |
| Convulsion | 2 (1.0) | 13 (0.7) | 0.658 |
| Confusion | 0 (0) | 28 (1.5) | 0.106 |
| Lethargy | 0 (0) | 14 (0.8) | 0.385 |
| Coma | 2 (1.0) | 16 (0.9) | 0.702 |
| Image findings | |  |  |
| Pericardial effusion | 3 (1.4) | 57 (3.1) | 0.268 |
| Pelvic effusion | 6 (2.9) | 11 (0.6) | 0.005 |
| Pleural effusion | 28 (13.4) | 263 (14.1) | 0.863 |
| Chest radiographic abnormality | 32 (15.3) | 533 (28.6) | <0.001 |
| Ascites | 12 (5.7) | 17 (0.9) | <0.001 |
| Splenomegaly | 53 (25.4) | 145 (7.8) | <0.001 |
| Hepatomegaly | 48 (23.0) | 49 (2.6) | <0.001 |

Data are n (%). NA, not applicable.

Pediatric patients, age 0–14 years; elderly patients, age ≥60 years.

All clinical symptoms (non-specific, skin, respiratory, gastrointestinal, hemorrhagic, and neurological) were reported before or on hospital admission, all abnormal image findings were reported after hospital admission.

p value calculated by use of χ^2^ test or Fisher's exact test between pediatric and elderly patients.

COPD, chronic obstructive pulmonary disease.

Hemorrhagic manifestations^*^, patients with one or more hemorrhagic symptoms.

Neurological manifestations^*^, patients with one or more neurological symptoms.
