# Supplementary material for: Clinical characteristics and risk factors for severe scrub typhus in pediatric and elderly patients
Source: PLoS Negl Trop Dis. 2022 Apr 29;16(4):e0010357. doi: 10.1371/journal.pntd.0010357 (PMC9053809; doi:10.1371/journal.pntd.0010357)
Supplement: S6 Table — NA, not applicable. The non-significant association was left blank. OR, odds ratio. CI, confidence interval. Pediatric patients, age 0–14 years; elderly patients, age ≥60 years. (DOCX) [file pntd.0010357.s006.docx]

**S6 Table: Association between clinical characteristics and severe scrub typhus by multivariate logistic regression analysis for pediatric and elderly patients.**

| **Variables** | **Pediatric patients (n=209)** | |  | **Elderly patients (n=1,865)** | |
| --- | --- | --- | --- | --- | --- |
|  | **Adjusted OR (95% CI)** | **p value** |  | **Adjusted OR (95% CI)** | **p value** |
| Age |  |  |  | 1.05 (1.03–1.07) | <0.001 |
| Time from symptom onset  to hospital admission |  |  |  | 1.06 (1.02–1.11) | 0.008 |
| Comorbidities |  |  |  |  |  |
| Cerebral infarction | NA | NA |  | 1.94 (1.09–3.45) | 0.024 |
| Non-specific manifestations |  |  |  |  |  |
| Peripheral edema | 38.99 (9.96–152.67) | <0.001 |  | 2.00 (1.11–3.60) | 0.020 |
| Icteric sclera |  |  |  | 4.13 (1.67–10.19) | 0.002 |
| Respiratory manifestations |  |  |  |  |  |
| Dyspnea | 33.71 (3.95–287.53) | 0.001 |  | 11.69 (7.33–18.64) | <0.001 |
| Gastrointestinal |  |  |  |  |  |
| Abdominal pain | 8.80 (2.08–37.27) | 0.003 |  |  |  |
| Vomit |  |  |  | 1.98 (1.27–3.11) | 0.003 |
| Hemorrhagic manifestations |  |  |  |  |  |
| Macroscopic hematuria |  |  |  | 6.89 (1.34–35.32) | 0.021 |
| Neurological manifestations |  |  |  |  |  |
| Confusion |  |  |  | 4.75 (1.87–12.04) | 0.001 |
| Lethargy |  |  |  | 5.19 (1.27–21.27) | 0.022 |
| Dysphoria | 19.30 (2.26–165.07) | 0.007 |  |  |  |

NA, not applicable. The non-significant association was left blank.

OR, odds ratio. CI, confidence interval.

Pediatric patients, age 0–14 years; elderly patients, age ≥60 years.
