# Supplementary material for: Clinical characteristics and risk factors for severe scrub typhus in pediatric and elderly patients
Source: PLoS Negl Trop Dis. 2022 Apr 29;16(4):e0010357. doi: 10.1371/journal.pntd.0010357 (PMC9053809; doi:10.1371/journal.pntd.0010357)
Supplement: S7 Table — Data was no./No. (%), no. is the number of patients with presence of laboratory abnormality. No. is the total number of patients with available data. Ranks of laboratory abnormalities according to their frequencies were listed for two age subgroups separately. Pediatric patients, age 0–14 years; elderly patients, age ≥60 years. p value calculated for the difference between pediatric patients and elderly patients by use of χ2 test or Fisher’s exact test. The normal range of laboratory indicators was expressed as that of the normal adult male. Normal ranges in different sex and age were shown in S8 Table. WBC, white blood cells; PLT, platelet; HGB, hemoglobin; LYM, lymphocyte; NEU, neutrophil; MON, monocyte; HCT, hematocrit; MCV, mean corpuscular volume; TBIL, total bilirubin; ALT, alanine aminotransferase; ALB, albumin; GLB, globulin; CREA, creatinine; BUN, blood urea nitrogen; CRP, C reactive protein. (DOCX) [file pntd.0010357.s007.docx]

**S7 Table****: The** **laboratory abnormalities on hospital admission compared between pediatric and elderly patients with scrub typhus.**

| **Laboratory indicators** | **Pediatric patients** | |  | **Elderly patients** | |  | **p value** |
| --- | --- | --- | --- | --- | --- | --- | --- |
|  | **Rank** | **no./No. (%)** |  | **Rank** | **no./No. (%)** |  |  |
| Hematological indicators |  |  |  |  |  |  |  |
| WBC count >10 (×10^9^/L) [4–10] | 11 | 46/194 (23.7) |  | 13 | 353/1,716 (20.6) |  | 0.354 |
| PLT count <100 (×10^9^/L) [100–300] | 4 | 108/194 (55.7) |  | 7 | 667/1,719 (38.8) |  | <0.001 |
| HGB <120 (g/L) [120–165] | 6 | 88/193 (45.6) |  | 8 | 590/1,699 (34.7) |  | 0.004 |
| LYM percent <20 (%) [20–40] | 8 | 68/193 (35.2) |  | 6 | 981/1,709 (57.4) |  | <0.001 |
| NEU percent >70 (%) [50–70] | 9 | 59/190 (31.1) |  | 5 | 1,071/1,694 (63.2) |  | <0.001 |
| MON percent >10 (%) [3–10] | 10 | 58/190 (30.5) |  | 14 | 247/1,648 (15) |  | <0.001 |
| HCT <40 (%) [40–50] | 7 | 88/194 (45.4) |  | 2 | 1,279/1,718 (74.4) |  | <0.001 |
| MCV <82 (FL) [82–100] | 3 | 144/193 (74.6) |  | 12 | 396/1,717 (23.1) |  | <0.001 |
| Biochemical indicators |  |  |  |  |  |  |  |
| TBIL >17.1 (umol/L) [5.1–17.1] | 14 | 16/171 (9.4) |  | 11 | 410/1,627 (25.2) |  | <0.001 |
| ALT >40 (U/L) [0–40] | 5 | 102/186 (54.8) |  | 3 | 1,246/1,717 (72.6) |  | <0.001 |
| ALB <35 (g/L) [35–55] | 1 | 152/171 (88.9) |  | 4 | 1,236/1,708 (72.4) |  | <0.001 |
| GLB <20 (g/L) [20–40] | 13 | 18/155 (11.6) |  | 15 | 38/1,441 (2.6) |  | <0.001 |
| CREA >106 (umol/L) [53–106] | 12 | 25/154 (16.2) |  | 9 | 466/1,696 (27.5) |  | 0.003 |
| BUN >7.1 (mmol/L) [3.2–7.1] | 15 | 4/59 (6.8) |  | 10 | 220/860 (25.6) |  | 0.002 |
| CRP >8.2 (mg/L) [0.07–8.2] | 2 | 145/182 (79.7) |  | 1 | 1,123/1,277 (87.9) |  | 0.003 |

Data was no./No. (%), no. is the number of patients with presence of laboratory abnormality. No. is the total number of patients with available data. Ranks of laboratory abnormalities according to their frequencies were listed for two age subgroups separately.

Pediatric patients, age 0–14 years; elderly patients, age ≥60 years.

p value calculated for the difference between pediatric patients and elderly patients by use of χ^2^ test or Fisher's exact test.

The normal range of laboratory indicators was expressed as that of the normal adult male. Normal ranges in different sex and age were shown in S8 Table.

WBC, white blood cells; PLT, platelet; HGB, hemoglobin; LYM, lymphocyte; NEU, neutrophil; MON, monocyte; HCT, hematocrit; MCV, mean corpuscular volume; TBIL, total bilirubin; ALT, alanine aminotransferase; ALB, albumin; GLB, globulin; CREA, creatinine; BUN, blood urea nitrogen; CRP, C reactive protein.
