# Supplementary material for: Clinical characteristics and risk factors for severe scrub typhus in pediatric and elderly patients
Source: PLoS Negl Trop Dis. 2022 Apr 29;16(4):e0010357. doi: 10.1371/journal.pntd.0010357 (PMC9053809; doi:10.1371/journal.pntd.0010357)
Supplement: S8 Table — WBC, white blood cells; PLT, platelet; HGB, hemoglobin; LYM, lymphocyte; NEU, neutrophil; MON, monocyte; HCT, hematocrit; MCV, mean corpuscular volume; TBIL, total bilirubin; ALT, alanine aminotransferase; ALB, albumin; GLB, globulin; CREA, creatinine; BUN, blood urea nitrogen; CRP, C reactive protein. (DOCX) [file pntd.0010357.s008.docx]

**S8 Table: Normal ranges of laboratory indicators by sex and age.**

| **Laboratory indicators** | **Pediatric patients (age 0–14 years)** | |  | **Adult patients (age ≥15 years)** | |
| --- | --- | --- | --- | --- | --- |
|  | **Female** | **Male** |  | **Female** | **Male** |
| Hematological indicators |  |  |  |  |  |
| WBC count (×10^9^/L) | 5–12 | 5–12 |  | 4–10 | 4–10 |
| PLT count (×10^9^/L) | 140–440 | 140–440 |  | 100–300 | 100–300 |
| HGB (g/L) | 105–145 | 105–145 |  | 110–150 | 120–165 |
| LYM percent (%) | 31–40 | 31–40 |  | 20–40 | 20–40 |
| NEU percent (%) | 40–60 | 40–60 |  | 50–70 | 50–70 |
| MON percent (%) | 1–8 | 1–8 |  | 3–10 | 3–10 |
| HCT (%) | 35–45 | 40–50 |  | 35–45 | 40–50 |
| MCV (FL) | 82–100 | 82–100 |  | 82–100 | 82–100 |
| Biochemical indicators |  |  |  |  |  |
| TBIL (umol/L) | 5.1–17.1 | 5.1–17.1 |  | 5.1–17.1 | 5.1–17.1 |
| ALT (U/L) | 0–50 | 0–50 |  | 0–40 | 0–40 |
| ALB (g/L) | 40–55 | 40–55 |  | 35–55 | 35–55 |
| GLB (g/L) | 20–40 | 20–40 |  | 20–40 | 20–40 |
| CREA (umol/L) | 18–62 | 18–62 |  | 44–97 | 53–106 |
| BUN (mmol/L) | 1.8–6.5 | 1.8–6.5 |  | 3.2–7.1 | 3.2–7.1 |
| CRP (mg/L) | 0–6 | 0–6 |  | 0.07–8.2 | 0.07–8.2 |

WBC, white blood cells; PLT, platelet; HGB, hemoglobin; LYM, lymphocyte; NEU, neutrophil; MON, monocyte; HCT, hematocrit; MCV, mean corpuscular volume; TBIL, total bilirubin; ALT, alanine aminotransferase; ALB, albumin; GLB, globulin; CREA, creatinine; BUN, blood urea nitrogen; CRP, C reactive protein.
