# Supplementary material for: Clinical characteristics and risk factors for severe scrub typhus in pediatric and elderly patients
Source: PLoS Negl Trop Dis. 2022 Apr 29;16(4):e0010357. doi: 10.1371/journal.pntd.0010357 (PMC9053809; doi:10.1371/journal.pntd.0010357)
Supplement: S9 Table — The non-significant association was left blank. OR, odds ratio; PLT, platelet; HGB, hemoglobin; TBIL, total bilirubin; ALT, alanine aminotransferase; CREA, creatinine; CI, confidence interval. Pediatric patients, age 0–14 years; elderly patients, age ≥60 years. The normal range of laboratory indicators was expressed as that of the normal adult male. Different normal ranges in different sex and age were shown in S8 Table. (DOCX) [file pntd.0010357.s009.docx]

**S9 Table: Association between laboratory measurements and severe scrub typhus by multivariate logistic regression analysis for pediatric and elderly patients.**

| **Variables** | **Pediatric patients** | |  | **Elderly patients** | |
| --- | --- | --- | --- | --- | --- |
|  | **Adjusted OR (95% CI)** | **p value** |  | **Adjusted OR (95% CI)** | **p value** |
| Age |  |  |  | 1.05 (1.02–1.09) | 0.002 |
| Time from symptom onset |  |  |  | 1.08 (1.01–1.16) | 0.026 |
| to hospital admission |  |  |  |  |  |
| PLT count <100 (×10^9^/L) | 6.03 (1.01–36.03) | 0.049 |  | 3.15 (1.92–5.16) | <0.001 |
| HGB <120 (g/L) | 13.22 (1.54–113.5) | 0.019 |  | 2.84 (1.76–4.60) | <0.001 |
| TBIL >17.1 (umol/L) | 6.40 (1.17–34.99) | 0.032 |  | 3.17 (1.97–5.11) | <0.001 |
| ALT >40 (U/L) | 10.53 (1.14–97.48) | 0.038 |  |  |  |
| CREA >106 (umol/L) |  |  |  | 2.44 (1.50–3.95) | <0.001 |

The non-significant association was left blank. OR, odds ratio; PLT, platelet; HGB, hemoglobin; TBIL, total bilirubin; ALT, alanine aminotransferase; CREA, creatinine; CI, confidence interval.

Pediatric patients, age 0–14 years; elderly patients, age ≥60 years.

The normal range of laboratory indicators was expressed as that of the normal adult male. Different normal ranges in different sex and age were shown in S8 Table.
