# Supplementary material for: Clinical characteristics and risk factors for severe scrub typhus in pediatric and elderly patients
Source: PLoS Negl Trop Dis. 2022 Apr 29;16(4):e0010357. doi: 10.1371/journal.pntd.0010357 (PMC9053809; doi:10.1371/journal.pntd.0010357)
Supplement: S10 Table — NA, not applicable. Coefficients were estimated that how many units of increase or decrease for major laboratory indicators in severe patients from symptom onset to laboratory test with mild patients group used as reference. PLT, platelet; HGB, hemoglobin; TBIL, total bilirubin; ALT, alanine aminotransferase; CREA, creatinine; CI, confidence interval. Pediatric patients, age 0–14 years; elderly patients, age ≥60 years. (DOCX) [file pntd.0010357.s010.docx]

**S10 Table: Estimated coefficients and statistical significance of major** **laboratory** **indicators associated with severe disease** **for pediatric patients and elderly patients during the days** **from symptom onset to laboratory test by using generalized estimating equation.**

| **Variables** | **Pediatric patients** | |  | **Elderly patients** | |
| --- | --- | --- | --- | --- | --- |
|  | **Coefficients (95% CI)** | **p value** |  | **Coefficients (95% CI)** | **p value** |
| PLT count (×10^9^/L) | -82.05 (-112.58, -51.52) | <0.001 |  | -51.22 (-58.05, -44.4) | <0.001 |
| HGB (g/L) | -17.68 (-21.31, -14.06) | <0.001 |  | -14.23 (-15.81, -12.65) | <0.001 |
| TBIL (umol/L) | 20.45 (9.07, 31.83) | <0.001 |  | 22.69 (18.59, 26.79) | <0.001 |
| ALT (U/L) | 5.80 (-8.67, 20.27) | 0.432 |  | NA | NA |
| CREA (umol/L) | NA | NA |  | 41.04 (31.35, 50.74) | <0.001 |

NA, not applicable. Coefficients were estimated that how many units of increase or decrease for major laboratory indicators in severe patients from symptom onset to laboratory test with mild patients group used as reference.

PLT, platelet; HGB, hemoglobin; TBIL, total bilirubin; ALT, alanine aminotransferase; CREA, creatinine; CI, confidence interval.

Pediatric patients, age 0–14 years; elderly patients, age ≥60 years.
