# Supplementary material for: Clinical characteristics and risk factors for severe scrub typhus in pediatric and elderly patients
Source: PLoS Negl Trop Dis. 2022 Apr 29;16(4):e0010357. doi: 10.1371/journal.pntd.0010357 (PMC9053809; doi:10.1371/journal.pntd.0010357)
Supplement: S11 Table — Data are n (%). Other antibiotics included minocycline, levofloxacin, ciprofloxacin, erythrocin and roxithromycin, etc. (DOCX) [file pntd.0010357.s011.docx]

**S11 Table:** **Therapy types for pediatric and elderly patients.**

| **Therapy types** | **Pediatric patients (n=202)** | **Elderly patients (n=1,735)** | **p value** |
| --- | --- | --- | --- |
| Doxycycline | 20 (9.9) | 1,125 (64.8) | <0.001 |
| Azithromycin | 83 (41.1) | 86 (5.0) | <0.001 |
| Chloramphenicol | 40 (19.8) | 135 (7.8) | <0.001 |
| Other antibiotics | 17 (8.4) | 130 (7.5) | 0.743 |
| Combined administration  of over 2 antibiotics | 33 (16.3) | 228 (13.1) | 0.250 |
| Supportive treatment | 9 (4.5) | 31 (1.8) | 0.030 |

Data are n (%).

Other antibiotics included minocycline, levofloxacin, ciprofloxacin, erythrocin and roxithromycin, etc.
