# Supplementary figures and images for: Clinical characteristics and risk factors for severe scrub typhus in pediatric and elderly patients
Source: PLoS Negl Trop Dis. 2022 Apr 29;16(4):e0010357. doi: 10.1371/journal.pntd.0010357 (PMC9053809; doi:10.1371/journal.pntd.0010357)

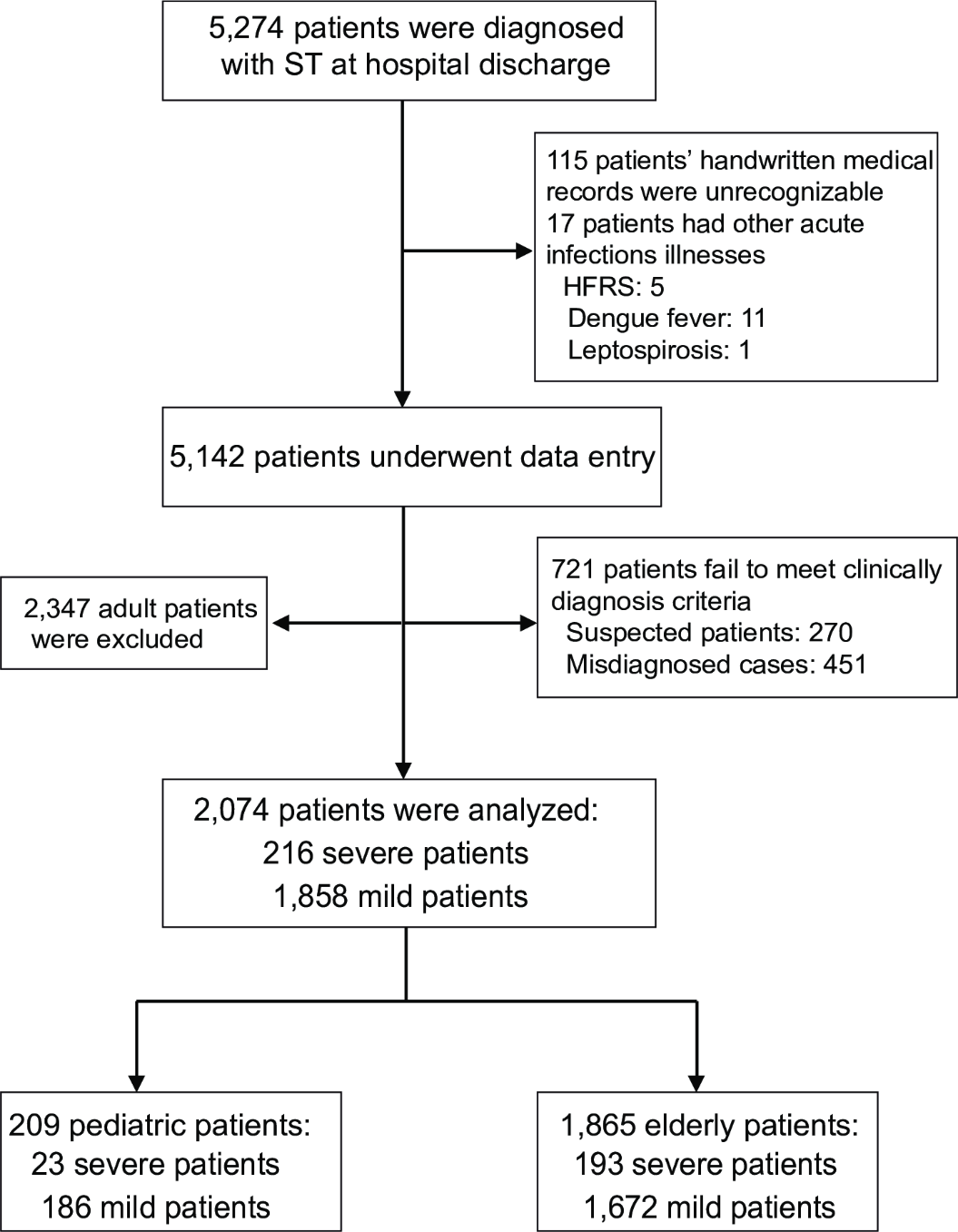

Supplement: S1 Fig — ST, scrub typhus. HFRS, hemorrhagic fever with renal syndrome. Pediatric patients, age 0–14 years; elderly patients, age ≥60 years. (TIF) [file pntd.0010357.s012.tif]

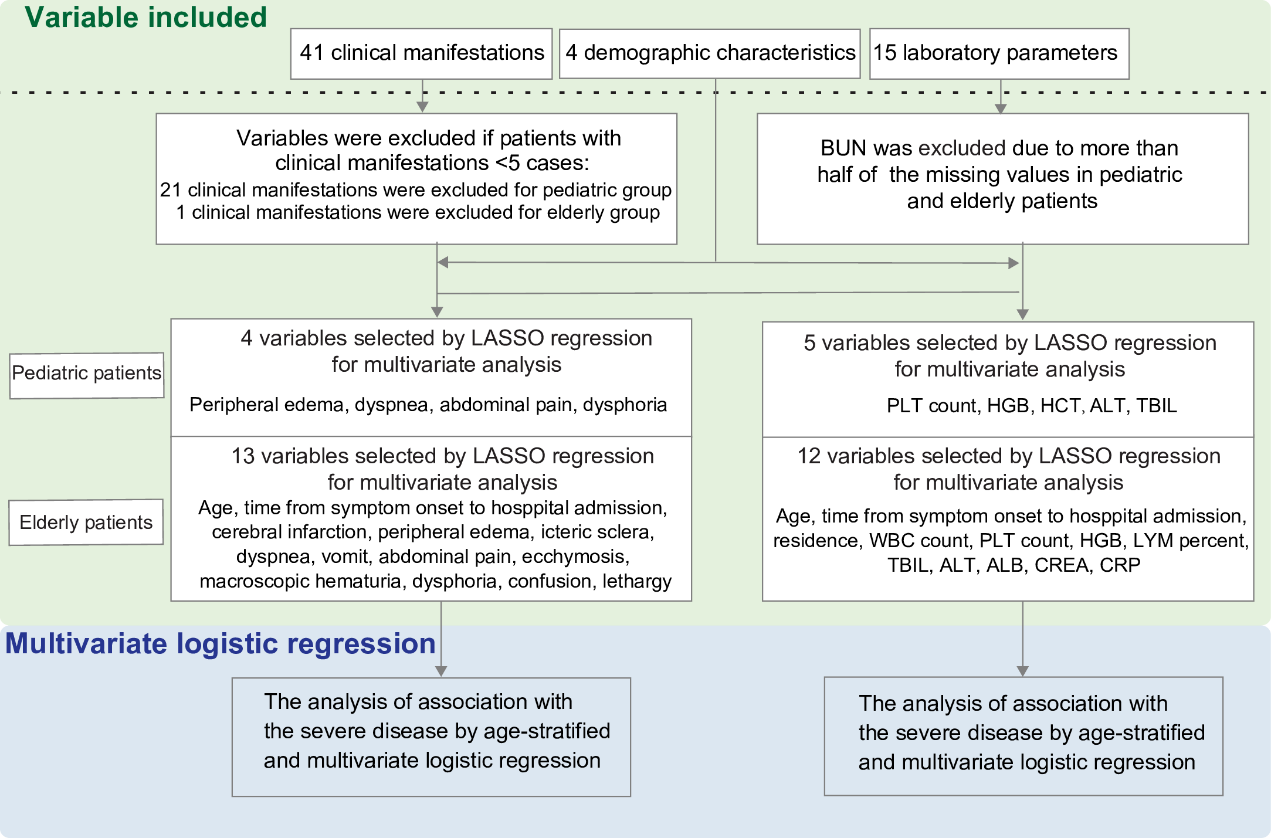

Supplement: S2 Fig — Demographic characteristics (age, sex, time from symptom onset to hospital admission, and residence). Clinical manifestations (hypertension, diabetes, coronary heart disease, cerebral infarction, viral hepatitis, COPD, smoking, drinking, fever, headache, dizziness, feeble, myalgias, arthralgia, lumbago, peripheral edema, lymphadenopathy, icteric sclera, skin rash, eschar, ulcer, cough, expectoration, enlarged tonsils, dyspnea, anorexia, nausea, vomit, abdominal pain, diarrhea, ecchymosis, petechiae, conjunctival hyperemia, gingival bleeding, melena, macroscopic hematuria, dysphoria, convulsion, confusion, lethargy, and coma). Laboratory parameters (WBC count, PLT count, HGB, LYM percent, NEU percent, MON percent, HCT, MCV, TBIL, ALT, ALB, GLB, CREA, BUN, and CRP). Variables were excluded if patients with clinical manifestations <5 cases: 21 clinical manifestations were excluded for pediatric patients (hypertension, diabetes, coronary heart disease, cerebral infarction, viral hepatitis, COPD, smoking, drinking, arthralgia, lumbago, icteric sclera, ecchymosis, petechiae, gingival bleeding, melena, macroscopic hematuria, convulsion, confusion, lethargy, coma, and fever was also excluded as present in all pediatric patients); gingival bleeding was excluded for elderly patients. BUN was excluded if missing values >50% in pediatric and elderly patients. COPD, chronic obstructive pulmonary disease. residence, area type of patients’ residence (rural or urban). Pediatric patients, age 0–14 years; elderly patients, age ≥60 years. WBC, white blood cells; PLT, platelet; HGB, hemoglobin; LYM, lymphocyte; NEU, neutrophil; MON, monocyte; HCT, hematocrit; MCV, mean corpuscular volume; TBIL, total bilirubin; ALT, alanine aminotransferase; ALB, albumin; GLB, globulin; CREA, creatinine; BUN, blood urea nitrogen; CRP, C reactive protein. (TIF) [file pntd.0010357.s013.tif]
